# Multi-Omics Analysis Revealed the *AGR-FC.C3* Locus of *Brassica napus* as a Novel Candidate for Controlling Petal Color

**DOI:** 10.3390/plants13040507

**Published:** 2024-02-11

**Authors:** Yiran Ding, Huaixin Li, Xinmin Liu, Xin Cheng, Wang Chen, Mingli Wu, Liurong Chen, Jianjie He, Hongbo Chao, Haibo Jia, Chunhua Fu, Maoteng Li

**Affiliations:** 1 Department of Biotechnology, College of Life Science and Technology, Huazhong University of Science and Technology, Wuhan 430074, China; dingyiran@hust.edu.cn (Y.D.); huaixin_lll@hust.edu.cn (H.L.); d202381021@hust.edu.cn (X.L.); d202381022@hust.edu.cn (X.C.); m202172334@hust.edu.cn (W.C.); d202280823@hust.edu.cn (M.W.); m202172254@hust.edu.cn (L.C.); jianjie_he@hust.edu.cn (J.H.); haibo.jia@hust.edu.cn (H.J.); 2 Key Laboratory of Molecular Biophysics of the Ministry of Education, Wuhan 430074, China; 3 School of Agricultural Sciences, Zhengzhou University, Zhengzhou 450001, China; chaohongbo@zzu.edu.cn

**Keywords:** petal color, carotenoid, bulked segregant analysis, differentially expressed genes

## Abstract

Variations in the petal color of *Brassica napus* are crucial for ornamental value, but the controlled loci for breeding remain to be unraveled. Here, we report a candidate locus, *AGR-FC.C3*, having conducted a bulked segregant analysis on a segregating population with different petal colors. Our results showed that the locus covers 9.46 Mb of the genome, harboring 951 genes. BnaC03.MYB4, BnaC03.MYB85, BnaC03.MYB73, BnaC03.MYB98, and BnaC03.MYB102 belonging to MYB TFs families that might regulate the petal color were observed. Next, a bulk RNA sequencing of white and orange-yellow petals on three development stages was performed to further identify the possible governed genes. The results revealed a total of 51 genes by overlapping the transcriptome data and the bulked segregant analysis data, and it was found that the expression of *BnaC03.CCD4* was significantly up-regulated in the white petals at three development stages. Then, several novel candidate genes such as *BnaC03.ENDO3*, *BnaC03.T22F8.180*, *BnaC03.F15C21.8*, *BnaC03.Q8GSI6*, *BnaC03.LSD1*, *BnaC03.MAP1Da*, *BnaC03.MAP1Db*, and *BnaC03G0739700ZS* putative to controlling the petal color were identified through deeper analysis. Furthermo re, we have developed two molecular markers for the reported functional gene *BnaC03.CCD4* to discriminate the white and orange-yellow petal colors. Our results provided a novel locus for breeding rapeseed with multi-color petals.

## 1. Introduction

*B. napus* is an important oilseed crop, supplying a primary source of edible vegetable oils and high-protein animal feeds [1,2,3]. The petal color is an essential indicator of agronomic traits in rapeseed, and flowers with rich colors have played a vital role in attracting insects and other pollinators for adequate pollination [4,5]. Additionally, the petal color in *B. napus* also holds ornamental value, and economically beneficial flowers with specific colors have been obtained [6,7,8,9].

Flower coloration is primarily determined by the content of carotenoids, flavonoids, and betalains [4]. Carotenoids are responsible for developing yellow, orange, and red flower colors [4,10]. The enzymes responsible for cleaving specific double-bond regions of carotenoids are known as carotenoid cleavage oxygenases (CCOs) or carotenoid-cleaving dioxygenases (CCDs) [5]. For instance, the *CmCCD4a* gene is expressed explicitly in chrysanthemum petals, degrading carotenoids into colorless compounds and changing petal color from yellow to white [11]. Similarly, the flesh was yellow due to carotenoid accumulation in the *PpCCD4* mutant of peaches [12]. Mutations in the *CrtR-b* gene, which explicitly expresses β-carotene hydroxylase in tomato flowers, significantly decrease carotenoid levels and result in white petals [13]. The carotenoid isomerase (CRTISO) is crucial in carotenoid synthesis by converting the prolycopene to all-*trans* lycopene [14]; it was found that the pro-lycopene was accumulated in the mutation of *CmCRTISO* and led to the petal color changing from yellow to light yellow in melon [15]. The function of *BrCRTISO1* has also been confirmed to control the yellowish color of petals in *B. rapa* [16]. A significant reduction in total carotenoid content resulted in a creamy-white phenotype in petals through the site-directed mutagenesis of *BnaCRTISO* in *B. napus* [17].

Flavonoids play an important role in forming petal color [18]. Anthocyanins (a subclass of flavonoids) are widespread in vascular plants and are responsible for various colors in flowers, such as orange, red, purple, and blue [4]. Manipulating or modifying genes that are associated with anthocyanin synthesis or its precursors has been successfully used in altering the petal color in several plant species, for example, in *Torenia hybrida*, *Petunia hybrida*, *Nicotiana tabacum*, and *Eustoma grandiflorum* (Raf.) Shinn [19,20,21,22,23]. In red-flowered rapeseed, various colors ranging from raspberry red to beige red and zinc yellow can be achieved via RNA interference to target the *anthocyanidin synthase* (*ANS*) *BnaA03.ANS* gene at different levels [24].

Other regulation factors have also been uncovered to influence petal color. In *B. juncea*, the stable storage of carotenoids was disrupted in the mutation of *BjA02.PC1* and *BjB04.PC2* (belonging to the esterase/lipase/thioesterase family of acyltransferases) led to a white-flowering phenotype [25]. Additionally, a specific rapeseed line with a pair of *Orychophragmus violaceus* chromosome M4 exhibited reddish petals [26], and further analysis showed that the *OvPAP2* transcript of *O. violaceus* is expressed specifically in rapeseed petals [27].

Bulked segregant analysis (BSA) is a commonly used method that identifies the molecular markers associated with a specific gene or genomic region [28]. For example, *CCD4*, which had functions for controlling the petal color, was identified by combining BSA and positional cloning in *B. napus* [29]. Additionally, a transcriptomic and metabolomic analysis revealed that the reduced lutein and zeaxanthin are the primary reason for the appearance of white-flowered rapeseed [29], and the related genes (*BnaA09.ZEP* and *BnaC09.ZEP*) that encoded zeaxanthin epoxidase (ZEP) were identified using BSA in *B. napus* [30].

The molecular mechanisms underlying flower coloration are still not fully understood. In this study, F_1_ was first obtained by hybridizing the *B. napus* with orange-yellow petals (YP) and white petals (WP). Then, an F_2_ segregation population with a series of petal colors was obtained. The extreme material pools with WP and YP in F_2_ were selected for BSA-Seq and RNA-Seq analyses, and the associated genomic region (AGR) of *AGR-FC.C3* that might control the petal colors and some key genes involved in carotenoid pathways were identified. These findings could enhance the breeding process of ornamental rapeseed and provide a theoretical foundation for future breeding with white and orange-yellow flowers in *B. napus*.

## 2. Results

### 2.1. BSA for Petal Colors in B. napus

**Figure 1:**
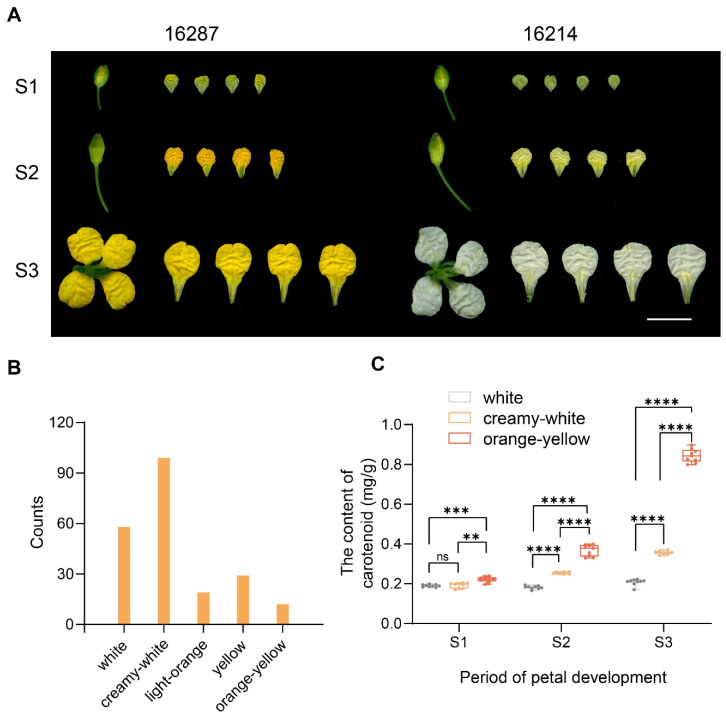
Phenotypic variations in orange-yellow and white flowers of *B. napus*. (**A**) Phenotypes of 16287 and 16214 in S1, S2, and S3. Bar, 1 cm. (**B**) The number of individuals with different petal colors in the F_1_ generation. (**C**) The determination of carotenoid content. Triplicate experiments with error bars represent a standard deviation. Nine biological replicates for each sample. ** *p* < 0.01, *** *p* < 0.001, **** *p* < 0.0001; ns, no significant.

The reciprocal hybridization between *B. napus* 16214 and 16287 was performed (Figure 1A). It was revealed that all F_1_ plants, no matter 16214 × 16287 or 16287 × 16214, had light-orange petals. The F_1_ seeds were subsequently sown in a field to yield the F_2_ generation. Further observation revealed that five distinct petal colors (white, creamy-white, light-orange, yellow, and orange-yellow) existed in the F_2_ generation (Figure 1B). Further analysis showed that the content of carotenoid in YP was significantly higher than in WP, particularly in S3, which was approximately three-fold more elevated than that of the WP (Figure 1C).

**Figure 2:**
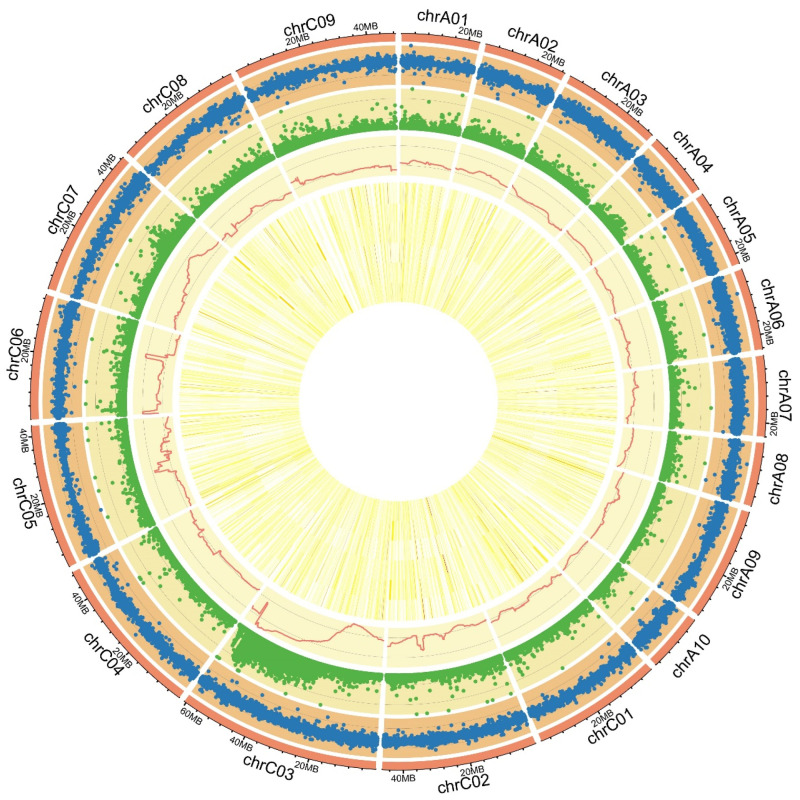
Detected InDels, SNPs, Δ(SNP-index) of BSA, and gene expression level of RNA-Seq. Among them, the 19 linkage groups are shown in the outermost circle. Four circles from the outside to the inside represent the InDels, SNPs, Δ(SNP-index), and gene expressions of YP-S1, YP-S2, YP-S3, WP-S1, WP-S2, and WP-S3, respectively.

**Figure 3:**
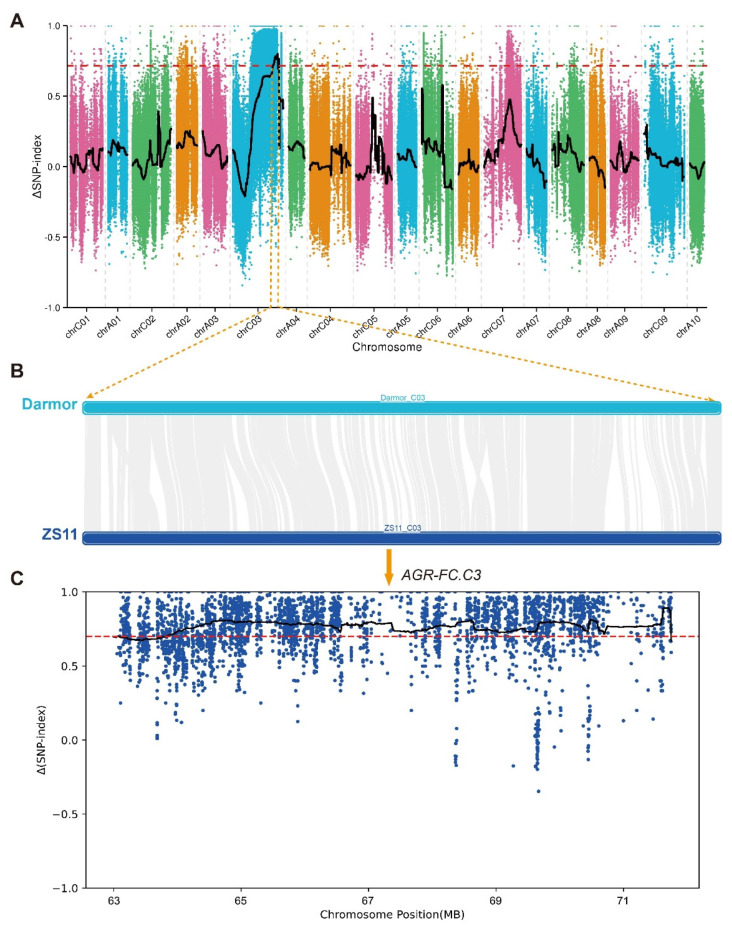
BSA analysis of WP and YP in *B. napus*. (**A**) Δ(SNP-index) of 19 chromosomes on the Darmor_*bzh* genome. (**B**) Alignment of the AGR hotspot region on the ChrC03 from the Darmor_*bzh* genome to the ZS11 genome. (**C**) Δ(SNP-index) of the AGR hotspot region on the ChrC03 of the ZS11 genome post mapping. The threshold is 0.715.

To investigate the genetic basis underlying the color difference, BSA was conducted on extreme pools with WP and YP in F_2_ lines, respectively. A total of 147.32 GB of clean data was obtained with a Q30 of 94.48% (Table S1). Each sample showed an average alignment rate of 93.50% to the reference genome, with an average 28× coverage depth and genome coverage of 94.90%, which satisfied the subsequent analysis requirements (Table S2). Genome-wide, a total of 668,993 single nucleotide polymorphisms (SNPs) and 668,993 insertion–deletions (InDels) between 16214 and 16287 were identified (Figure 2, Tables S3 and S4). It was revealed that a significant genomic region of 9.46 Mb (63.03–72.49 Mb) on ChrC03 was associated with petal color (Figure 2 and Figure 3A). Since the Darmor_*bzh* genome has a gap in this region, this region in the Darmor_*bzh* was also mapped to the genome of Zhongshuang 11 (ZS11) (Figure 3B). Further analysis showed that a significantly associated region called *AGR-FC.C3* with a Δ(SNP-index) > 0.715 and *p*-value < 0.01 within this genomic region was detected (Figure 3C).

A total of 951 genes were found within the interval of *AGR-FC.C3*, and 71 were classified as TFs, in which the bHLH, Dof, MYB, and bZIP families were found to be the most abundant (Figure S1). TFs BnaC03.MYB4, BnaC03.MYB73, BnaC03.MYB98, BnaC03.MYB102, and BnaC03.MYB85, which regulate the biosynthesis of carotenoids and flavonoids, were observed, which might play important roles in petal color formation (Table S5). Additionally, the 951 genes within the ChrC03 interval were performed the Kyoto Encyclopedia of Genes and Genomes (KEGG) analysis, and several pathways, including the cysteine and methionine metabolism, RNA polymerase, taurine, and hypotaurine metabolism, and the citrate cycle were enriched (Figure S2).

### 2.2. RNA-Seq and Differentially Expressed Genes (DEGs) Analysis between the YP and WP in B. napus

To elucidate the underlying mechanisms responsible for color variation in petals, the YP and WP of S1, S2, and S3 were subjected to transcriptomic analysis. A total of 509.62 Gb of raw reads were obtained, with all samples exhibiting Q30 scores exceeding 93% (Table S6). The quality-controlled clean reads were then aligned to the ZS11 genome, with each sample achieving a mapping ratio exceeding 83% (Table S7). The expression levels (log_10_(FPKM+1)) of each sample were mainly distributed between 0 and 1 (Figure S3). Pearson correlation coefficients indicated a significant correlation between YP and WP at the same developmental stage (Figure S4A). Substantial differences were observed between petals at different developmental stages (Figure S4A). Principal component analysis (PCA) further revealed differences between YP-S3 and WP-S3. In contrast, no significant differences were observed during S1 and S2 (Figure S4B). Six DEGs were randomly selected for reverse transcription quantitative real-time PCR (RT-qPCR) analysis to verify the accuracy of the RNA-Seq. The consistent expression tendency existed with the transcriptomic results (Figure S5).

**Figure 4:**
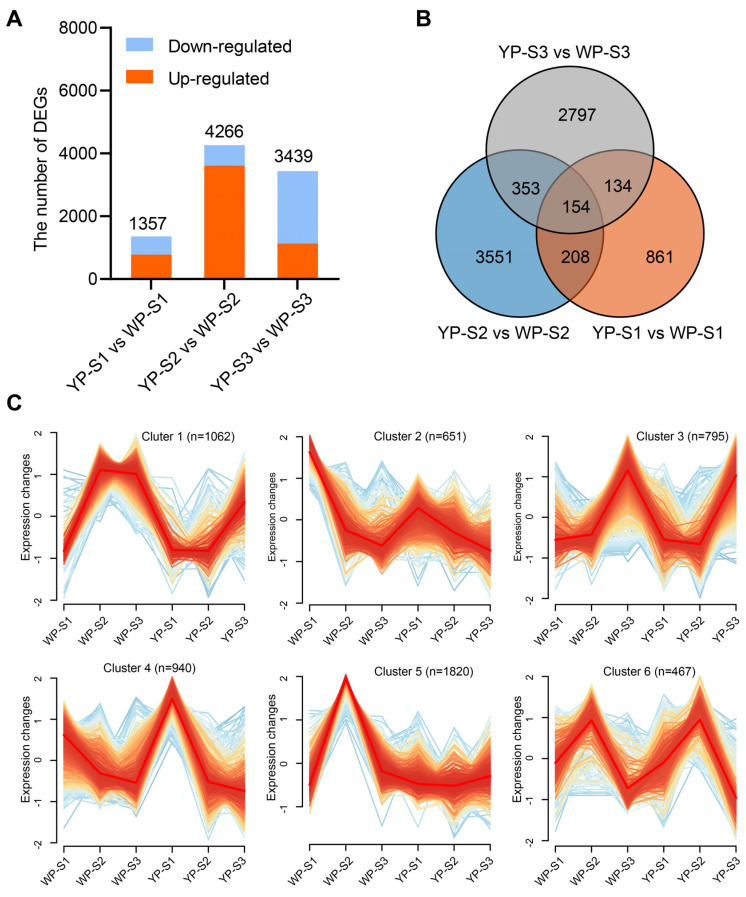
Analysis of differentially expressed genes between WP and YP. (**A**) The count of DEGs in the “YP-S1 vs. WP-S1”, “YP-S2 vs. WP-S2”, and “YP-S3 vs. WP-S3” groups. (**B**) Venn diagram of the three groups: “YP-S1 vs. WP-S1”, “YP-S2 vs. WP-S2”, and “YP-S3 vs. WP-S3”. (**C**) Trend plot showing the expression level changes in all DEGs in the “YP-S1 vs. WP-S1”, “YP-S2 vs. WP-S2”, and “YP-S3 vs. WP-S3” groups. n represents the number of genes in the cluster.

The clean data in YP and WP during S1, S2, and S3 (groupings named “YP-S1 vs. WP-S1”, “YP-S2 vs. WP-S2”, and “YP-S3 vs. WP-S3”) were used for the DEGs analysis. In the group “YP-S1 vs. WP-S1”, if the expression level of DEGs was higher in WP-S1 than that in YP-S1, that means the DEGs were up-regulated. It was revealed that in S2, the most significant number of DEGs (4266 genes) was detected in the “YP-S2 vs. WP-S2” group, and the up-regulated DEGs were approximately six-fold than that of the down-regulated DEGs (Figure 4A). However, the number of down-regulated DEGs was twice that of up-regulated DEGs in S3 (Figure 4A). In S1, the KEGG pathways of the inositol phosphate metabolism, tropane, piperidine and pyridine alkaloid biosynthesis, tyrosine metabolism, propanoate metabolism, glutathione metabolism, photosynthesis, and ABC transporters were highly enriched (Figure S6A). In S2, 4266 DEGs were significantly enriched in the proteasome, ascorbate and aldarate metabolism, inositol phosphate metabolism, phosphatidylinositol signaling system, N-glycan biosynthesis, SNARE interactions in vesicular transport, galactose metabolism, and ether lipid metabolism (Figure S6B). While in S3, more KEGG pathways were enriched, such as the peroxisome, DNA replication, photosynthesis, mismatch repair, glyoxylate and dicarboxylate metabolism, fatty acid elongation, zeatin biosynthesis, nucleotide excision repair, ABC transporters, phenylalanine metabolism, and autophagy (other) (Figure S6C). A Venn diagram showed that 154 DEGs were detected in all stages (Figure 4B), and 107, 110, and 117 DEGs were down-regulated in WP-S1, WP-S2, and WP-S3, respectively (Figure S7).

A total of 8058 DEGs obtained in S1, S2, and S3 were performed for trend analysis, and six significant trends were found. Interestingly, regardless of the color of the petals, whether orange-yellow or white, the expression patterns were identical in cluster 2, cluster 4, and cluster 6 (Figure 4C). The gene expression levels in cluster 2 were relatively higher in S1 and gradually decreased with the petal’s development. However, somewhat higher expression levels were observed in WP-S1, which indicated a gradual decline in gene expression with the petal’s development (Figure 4C). Similarly, the gene expression trend in cluster 4 resembled that of cluster 2, except higher expression levels were observed in YP-S1. Moreover, the gene expression trend in cluster 6 was consistent in both the WP and YP of S1, S2, and S3 (Figure 4C).

### 2.3. Overlapping Analysis of BSA and RNA-Seq Results

**Figure 5:**
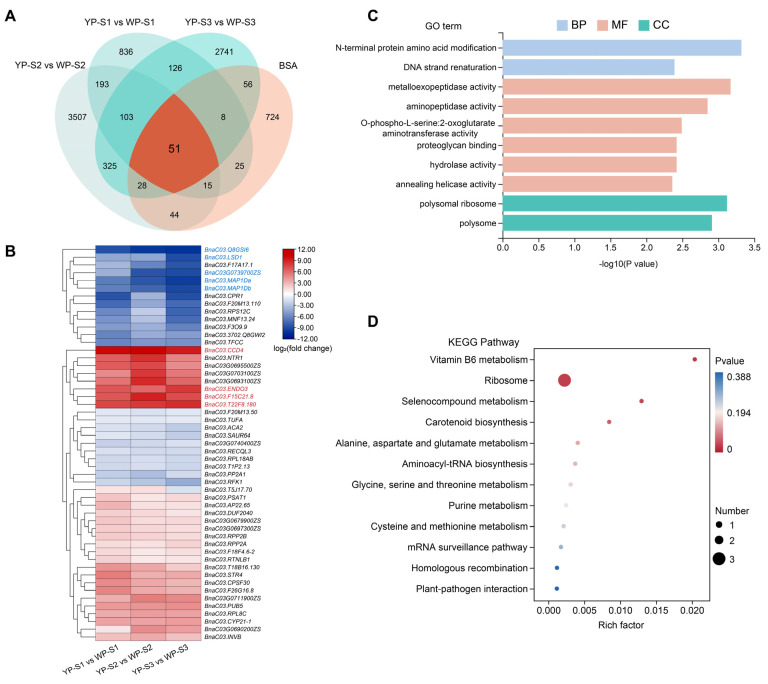
Combined analysis of BSA and transcriptome. (**A**) Venn diagram illustrating genes from significant regions on the ChrC03 selected through BSA and DEGs in the “YP-S1 vs. WP-S1”, “YP-S2 vs. WP-S2”, and “YP-S3 vs. WP-S3” groups. (**B**) Heatmap of 51 common genes with the fold change. Red indicates up-regulated, and blue indicates down-regulated. GO (**C**) and KEGG (**D**) enrichments of the 51 common genes.

To identify the key genes affecting the petal color, the genes obtained from the BSA were overlapped with the DEGs obtained via RNA-Seq, and 51 candidate genes that might be associated with petal color variation were identified (Figure 5A). Of these 51 genes, 23 and 27 were down-regulated and up-regulated in the WP-S1, WP-S2, and WP-S3, respectively (Figure 5B). One gene was up-regulated in WP-S1 and WP-S2, while it was down-regulated in WP-S3. Further analysis showed that *BnaC03.CCD4* (*BnaC03G0710000ZS*), *BnaC03.ENDO3* (*BnaC03G0721300ZS*), *BnaC03.T22F8.180* (*BnaC03G0714800ZS*), and *BnaC03.F15C21.8* (*BnaC03G0704000ZS*) were all up-regulated in WP-S1, WP-S2, and WP-S3. Moreover, *BnaC03.Q8GSI6* (*BnaC03G0714100ZS*), *BnaC03.LSD1* (*BnaC03G0719100ZS*), *BnaC03.MAP1Da* (*BnaC03G0690400ZS*), *BnaC03.MAP1Db* (*BnaC03G0690500ZS*), and *BnaC03G0739700ZS* were all down-regulated in WP-S1, WP-S2, and WP-S3 (Figure 5B).

A Gene Ontology (GO) enrichment analysis of these 51 candidate genes revealed that the significantly enriched terms were related to N-terminal protein amino acid modification, metalloexopeptidase activity, and polysomal ribosome (Figure 5C). *BnaC03.MAP1Da* and *BnaC03.MAP1Db* were enriched in the N metabolism and metalloexopeptidase activity, while *BnaC03.RPL18AB*, *BnaC03.RPS12C*, and *BnaC03.RPL8C* were enriched in polysomal ribosome. Moreover, a KEGG enrichment analysis indicated that the significantly enriched pathways were the Vitamin B6 metabolism, ribosome biogenesis, selenocompound metabolism, and carotenoid biosynthesis (Figure 5D).

Among these 51 candidate genes, the gene with the largest expression difference between the YP and WP was *BnaC03.CCD4* (in cluster 1), it was shown that the expressions in WP-S1, WP-S2, and WP-S3 were 1099, 1312, and 633 folds higher those in YP-S1, YP-S2, and YP-S3, respectively (Figure 5B). In cluster4, *BnaC03.LSD1* was a gene containing zinc-finger motifs and positively regulated the downstream genes as a TF; it was shown that the expressions in YP-S1, YP-S2, and YP-S3 were 20, 21, and 492 folds higher than those of the WP. It was also revealed that the expression of *BnaC03.MAP1Da* (in cluster 4) was down-regulated by 94, 352, and 851 folds in WP-S1, WP-S2, and WP-S3 compared to those of YP-S1, YP-S2, and YP-S3, respectively; similarly, *BnaC03.MAP1Db* (in cluster 4) was also down-regulated 85, 204, and 581 folds in WP-S1, WP-S2, and WP-S3 compared to those of YP-S1, YP-S2, and YP-S3, respectively. *BnaC03.Q8GSI6* (in cluster 4) was down-regulated 441, 1041, and 1233 folds in WP-S1, WP-S2, and WP-S3 compared to those in YP-S1, YP-S2, and YP-S3, respectively. In cluster 5, the expression of *BnaC03.ENDO3* was up-regulated in WP-S1, WP-S2, and WP-S3 with fold changes of 121, 70, and 265. In cluster 2, *BnaC03.T22F8.180* and *BnaC03.F15C21.8* might also positively regulate the formation of WP. In addition, nine DEGs (*BnaC03G0739700ZS*, *BnaC03G0740400ZS*, *BnaC03G0690200ZS*, *BnaC03G0697300ZS*, *BnaC03G0679900ZS*, *BnaC03G0711900ZS*, *BnaC03G0703100ZS*, *BnaC03G0693100ZS*, and *BnaC03G0695500ZS*) were annotated as unknown function, which might be the potential genes involved in petal color formation (Figure 5B).

### 2.4. The Carotenoid Pathway Influences the Petal Colors in B. napus

**Figure 6:**
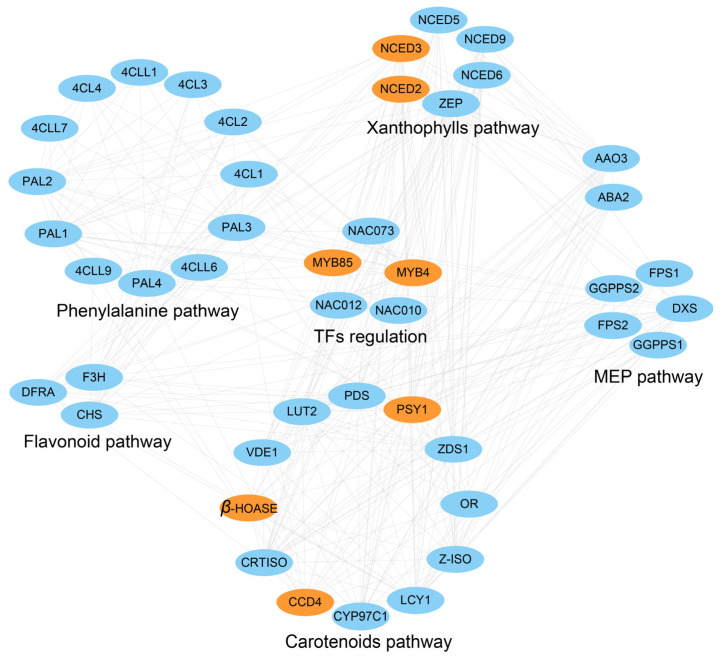
The network of DEGs and related pathways of phenylalanine, flavonoid, carotenoids, MEP, xanthophyll, and TF regulations. Orange ovals represent genes that are differentially expressed in WP and YP.

Based on the analysis of WP and YP, the genes related to TFs, the phenylalanine pathway, carotenoid pathway, flavonoid pathway, 2-C-methyl-D-erythritol 4-phosphate (MEP) pathway, and xanthophylls pathway were investigated to construct a regulation network. MYB4 and MYB85 might be at the center of regulating petal color by affecting the phenylalanine, carotenoid, and flavonoid pathways (Figure 6). BnaC03.MYB4 and BnaC03.MYB85 were located in the interval of *AGR-FC.C3* and were considered valuable candidates. In the network, it was shown that MYB4 and MYB85 might regulate the expressions of CCD4, nine-cis-epoxy carotenoid dioxygenase2 (NCED2), nine-cis-epoxy carotenoid dioxygenase3 (NCED3), beta-carotene hydroxylase (*β*-HOASE), and phytoene synthase 1 (PSY1) to influence petal color (Figure 6).

**Figure 7:**
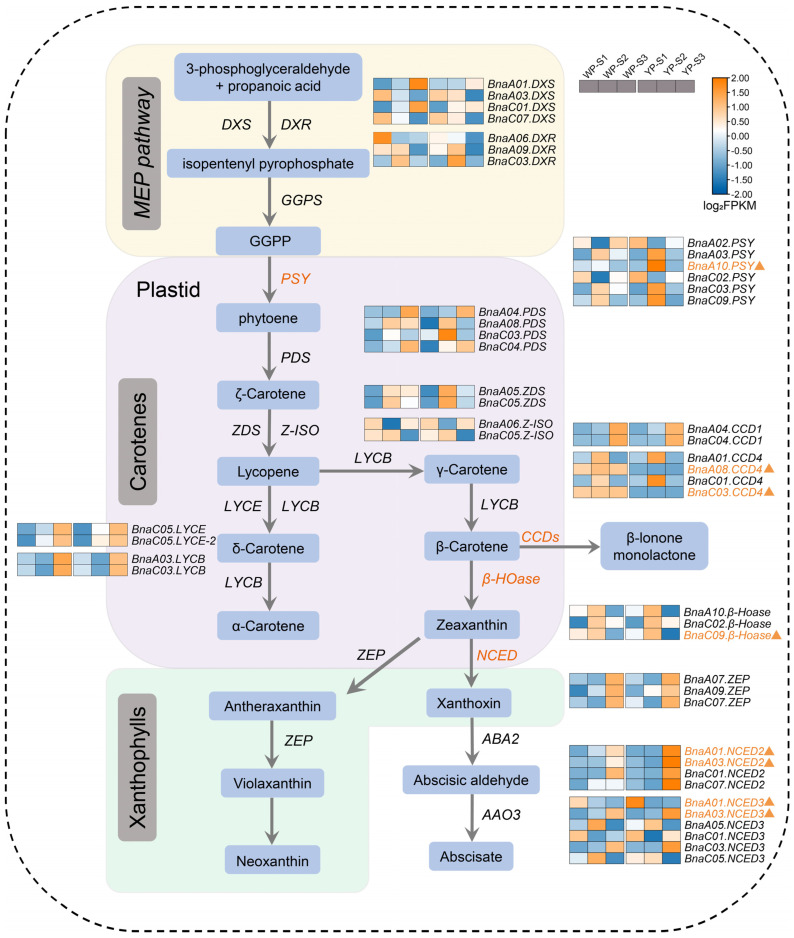
The biosynthesis pathway of carotenoid. This figure depicts the MEP pathway. The compounds produced within the carotenoid pathway are represented in blue rounded rectangles, while enzymes catalyzing the reactions are italicized. The samples from left to right represent WP-S1, WP-S2, WP-S3, YP-S1, YP-S2, and YP-S3. The color bar of the heatmap indicates log_2_FPKM, with FPKM values being average. The orange indicates high expression, while the blue indicates low expression. The orange triangles in the heatmap represent genes showing differential expressions in YP and WP. GGPP, geranylgeranyl diphosphate. Enzymes: DXS, 1-deoxy-d-xylulose 5-phosphate synthase; DXR, 1-deoxy-D-xylulose 5-phosphate reductoisomerase; PSY, phytoene synthase; PDS, phytoene desaturase; ZDS, zeta-carotene desaturase; Z-ISO, 15-cis-zeta-carotene isomerase; LYCE, lycopene cyclase; LYCB, lycopene beta/epsilon cyclase; *β*-Hoase, beta-carotene hydroxylase; ZEP, zeaxanthin epoxidase; CCD1, carotenoid cleavage dioxygenase 1; CCD4, carotenoid cleavage dioxygenase 4; NCED2, nine-cis-epoxycarotenoid dioxygenase 2; NCED3, nine-cis-epoxycarotenoid dioxygenase.

Previous studies have reported that the carotenoid pathway plays a crucial regulatory role in petal color formation. Therefore, the expressions of carotenoid pathway genes in YP and WP were analyzed in this study. This revealed the alterations in the expression of multiple genes encoding enzymes involved in carotenoid biosynthesis (Figure 7). The PSY can promote the geranylgeranyl diphosphate (GGPP) synthesis of phytoene, and it was shown that the *BnaA10.PSY* (*BnaA10G0196500ZS*) was up-regulated in YP-S2, which means more phytoene may be synthesized in YP. Multiple DEGs were found in the β-carotene degradation pathway, such as the expressions of *BnaA08.CCD4* (*BnaA08G0113600ZS*) and *BnaC03.CCD4* in WP being much higher than those of YP in S1, S2, and S3, suggesting that the carotenoid degradation was the reason for the petal color turning from yellow to white. *BnaC09.β-Hoase* (*BnaC09G0345900ZS*) could inhibit the conversion of β-carotene to zeaxanthin, and it was found to be down-regulated in YP-S3. Furthermore, the expression of NCED involved in zeaxanthin generation was also detected; *BnaA01.NCED2* (*BnaA01G0092600ZS*) was down-regulated in YP-S2 and up-regulated in YP-S3, while *BnaA03.NCED2* (*BnaA03G0447100ZS*) and *BnaA03.NCED3* (*BnaA03G0338300ZS*) were up-regulated in YP-S3, and *BnaA01.NCED3* (*BnaA01G0353200ZS*) was upregulated in YP-S2 (Figure 7). In addition, no obvious DEGs related to the flavonoid pathway of *BnaANS*, *dihydroflavonol-4-reductase* (*BnaDFR*), *flavanone 3-hydroxylase* (*BnaF3H*), *UDP-glycosyltransferase* (*BnaUFGT*), and *UDP-glucose: flavonoid 3-o-glucosyltransferase* (*BnaUF3GT*) were detected in the YP or WP (Figure S8).

### 2.5. Application of Linkage Marker of BnaC03.CCD4 in B. napus

The SNPs between *BnaC03.CCD4* in the WP and YP were further compared, and several SNPs in the exon region were found, in which eight SNPs were found in the second exon (including two C-to-T changes, two G-to-A changes, two A-to-T changes, and A-to-G and C-to-G changes) (Figure S9).

**Figure 8:**
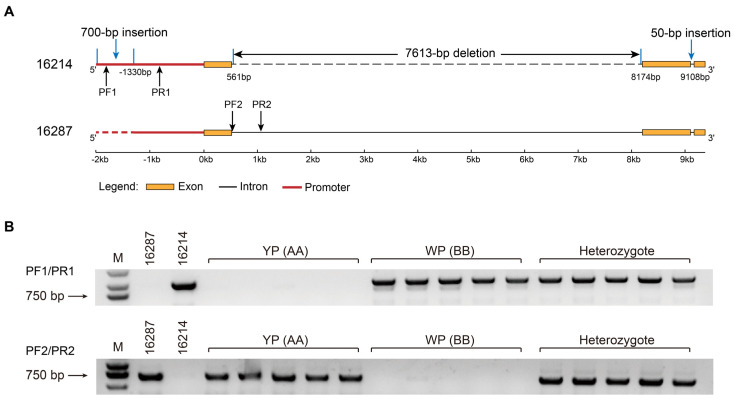
Allelic variations in *BnaC03.CCD4* and molecular maker design. (**A**) Sequence variation in the *BnaC03.CCD4* of 16214 and 16287. The primers PF1/PR1 were designed in the promoter region of 16214. The primers PF2/PR2 were designed in the intron region of 16287. (**B**) Confirmation of co-segregated maker (16214.PF1/16214.PR1; 16287.PF2/16287.PR2) in 16214, 16287, and the F_2_ population with 217 individuals derived from 16214 × 16287. M, marker.

To further investigate the sequence variations in *BnaC03.CCD4* between 16214 and 16287, the whole gene length sequence of *BnaC03.CCD4* was amplified from the genomic DNA for Sanger sequencing. A 7613 bp deletion and 50 bp insertion were identified in the first and second intron of *BnaC03.CCD4* in 16214, which might influence the mRNA splicing (Figure 8A). A 700 bp insertion was detected in the promoter of *BnaC03.CCD4* in 16214, resulting in a higher expression in WP than that in YP (Figure 8A, Figure S5).

To rapidly identify *B. napus* individuals with different petal colors, a pair of primers was designed according to the sequence variations in *BnaC03.CCD4* between 16214 and 16287, which could act as molecular markers (Figure 8B, Table S8). A band with 951 bp was found in 16214, the WP homozygote (BB), and the heterozygote when using primers of PF1/PR1, while no band was found in 16287 and the YP homozygote (AA) (Figure 8B). When using primers of PF2/PR2, a band with 695 bp was found in 16287, the YP homozygote (AA), and the heterozygote, while no band in 16214 and the WP homozygote (BB) (Figure 8B). The molecular markers of 16214.PF1/16214.PR1 and 16287.PF2/16287.PR2 were implied to distinguish the WP and YP (Figure 8B, Table S8).

## 3. Discussion

Flower color is one of the most critical traits in *B. napus*, and many researchers have attempted to explore the genetic mechanisms of flower color formation in recent years [25,29,30,31]. However, the molecular mechanisms affecting flower color are still insufficient.

To further understand the regulatory mechanism of petal color, QTL, BSA, transcriptomics, and metabolomics have been used to identify the key factors that affect the petal color formation previously. The candidate gene of *BnaPAP2.A7b*, located within 219.81 kb on chromosome A07, was identified through a combination of BSA and QTL fine mapping in *B. napus* [32], and the ectopic expression of the *OvPAP2* in *Orychophragmus violaceus* could form red-flowered rapeseed [27]. It was found that a 211 bp insertion in the promoter of *BnaPAP2.A7b* could cause a change in flower color from red to orange in rapeseed [32]. In this study, a significant locus, *AGR-FC.C3* on ChrC03, which covers 9.46 Mb, was obtained through a BSA on WP and YP. Moreover, the number of DEGs between the WP and YP varied greatly in different developmental stages. A co-analysis of BSA and transcriptomic results showed that 51 genes were obtained, as well as some candidate genes, such as *BnaC03.ENDO3*, *BnaC03.T22F8.180*, *BnaC03.F15C21.8*, *BnaC03.Q8GSI6*, *BnaC03.LSD1*, *BnaC03.MAP1Da*, *BnaC03.MAP1Db*, and *BnaC03G0739700ZS* that have not been reported to be associated with flower color. *BnaC03.ENDO3* is orthologous to the Arabidopsis gene *ENDONUCLEASE 3*, which encodes a putative endonuclease and expressed in the floral meristem and stamen development; this gene positively regulates the anthocyanin metabolism [33]. *BnaC03.T22F8.180* is orthologous to the Arabidopsis gene *AT4G39280*, which is encoded in the phenylalanyl-tRNA synthetase [34]. *BnaC03.F15C21.8* is orthologous to the Arabidopsis gene *AT1G36280*, which encodes an L-Aspartase-like family protein [35]. *BnaC03.Q8GSI6* is a TRAM, LAG1, and CLN8 (TLC) lipid-sensing domain-containing protein, which is orthologous to Arabidopsis gene *AT4G19645*. However, there are no reports about the gene *BnaC03.Q8GSI6* and its Arabidopsis orthologous gene *AT4G19645*. *BnaC03.MAP1Da* and *BnaC03.MAP1Db*, orthologous to *AT4G37040*, encode a methionine aminopeptidase, and are enriched in the N metabolism and metalloexopeptidase activity [36]. *BnaC03.LSD1*, orthologous to *AT4G20380*, contains zinc-finger motifs and negatively regulates a basal defense pathway [37]. These results indicated the complex pathways for flower color regulation in *B. napus*.

Carotenoids have been considered one of the main factors that affect petal colors [17,31,38]. The previous research showed that the total carotenoid content in yellow-flowered rapeseed was much higher than that in the white-flowered inbred lines [39]. In this study, KEGG analysis also indicated that the carotenoid biosynthesis was enriched, and the total content of carotenoids in the YP was significantly higher than that in WP at the S2 and S3 stages, consistent with the previous studies [17,38]. The co-analysis of metabolomic and transcriptomic results revealed that γ-carotene might be the substance that influenced the pigment deposition in petals of *Liriodendron tulipifera*, and it indicated that the CRTISO and ε-lycopene cyclase (ε-LCY) were the core enzymes that encoded the specific orange pigment deposition in petals [40]. However, in the present study, the expression of genes that encode the CRTISO and ε-LCY enzymes between the WP and YP has not changed. Through map-based cloning, it was shown that the absence of *BnaC09.ZEP* and a 1695 bp deletion in *BnaA09.ZEP* could lead to a flower color change from yellow to orange [30]. It was also shown that the mutation of *BnaCRTISO* could cause significant down-regulations in the essential genes of *BnaPSY* and *BnaC4H* that are involved in carotenoid and flavonoid synthesis. Still, the expressions of some other genes that relate to carotenoid and xanthophyll synthesis (such as *BnaPDS3*, *BnaZEP*, and *BnaBCH1*) were up-regulated [17]. In the present study, the expression of *BnaA10.PSY* was up-regulated in YP-S2, which could lead to a higher production of phytoene through the promotion of carotenoid synthesis.

CCD4 has been shown to play an important role in flower color formation [11,29,38]. It was found that the *BnaC03.CCD4* is preferentially expressed in the petals of white-flowered rapeseed, and a CACTA-like transposable element 1 (TE1) insertion into its coding region led to the reduced expression in yellow-flowered rapeseed [29]. In the present results, we found that the *BnaC03.CCD4* is detected in the interval of *AGR-FC.C3*, and the expression in the WP was significantly higher than that in YP. The highly expressed *BnaC03.CCD4* could promote the degradation of carotenoid in petals, and then result in a white flower color. In addition to CCD4, other studies reported that NCED also plays an important role in flower color formation [39,41]. It was shown that the expression of *BnNCED4b* in white petals is significantly higher than that in yellow petals in rapeseed, which indicated that it might also play an important role in white petal formation [39]. The present study shows no difference in the NCED between WP-S1 and YP-S1. However, in S2, *BnaA01.NCED2* was down-regulated in the WP, while *BnaA01.NCED3* was up-regulated in WP. In S3, *BnaA01.NCED2*, *BnaA03.NCED2*, and *BnaA03.NCED3* were up-regulated in WP. The expression profiles of *BnaA01.NCED3*, *BnaA03.NCED2*, and *BnaA03.NCED3* were consistent with those in the previous report [41].

Eight SNPs were found to exist in the second exon of *BnaC03.CCD4* in the WP and YP, these variations might lead to the transformation from WP to YP. In addition, compared to 16214 with white petals, a completely different promoter region with a 700 bp insertion of *BnaC03.CCD4* was identified. Interestingly, a 7613 bp deletion and 50 bp insertion in the intron of *BnaC03.CCD4* were also detected in 16214. These results indicated that the different promoters of *BnaC03.CCD4* might promote its expression in 16214 and lead to the degradation of carotenoids. It was found that the CmGATA4 could lead to the low expression of *CmCCD4a-5* by directly binding to the promoter of *CmCCD4a-5* and then promoting the accumulation of carotenoids in the orange-flowered chrysanthemum mutant [38]. A pair of molecular markers were then successfully developed based on the allelic variation of *BnaC03.CCD4*, providing an ideal molecular marker for flower color breeding in *B. napus*. 

The flavonoid pathway is also involved in the formation of petal color [18,24,42], and it was reported that the flavonols, flavones, and anthocyanidins were the main contributors to petal coloration [42]. A KEGG functional annotation and RT-qPCR analysis revealed that the expression of *BnaA10g23330D* (*BnF3′H*) could affect the synthesis of downstream peonidin and delphinidin in *B. napus* [18]. The RNA-Seq analysis showed that genes involved in the anthocyanin biosynthesis pathway were primarily regulated by ANS, DFR, and UF3GT in *B. napus* petals [24]. The genetic analysis showed that the yellowish-white trait of *B. napus* was controlled by a single recessive gene, *BnaA08.PDS3*, and truncating this gene could lead to a reduction in carotenoid biosynthesis [31]. In the present study, no significant changes were found in *BnaANS*, *BnaDFR*, *BnaF3H*, *BnaUFGT*, and *BnaUF3GT* (the critical genes of the flavonoid pathway).

The MYBs that regulate the anthocyanin metabolism are mainly R2R3-MYB and R3-MYB [32]. R2R3-MYB-like are widely involved in a variety of primary and secondary metabolic processes in the plant phenylpropane-flavonoid metabolic pathway [27,43], which play an important role in the formation of final color in tissues and organs [44]. The *BnaA07.PAP2^In-184-317^* activates the expressions of genes related to anthocyanin synthesis and promotes the accumulation of anthocyanins in rapeseed petals [43]. Some novel TFs, such as BnaC03.MYB4, BnaC03.MYB73, BnaC03.MYB98, BnaC03.MYB102, and BnaC03.MYB85 that belong to MYB TF families were found in the present research. MYB4 and MYB85 might co-regulate the expressions of genes in the carotenoid pathway, such as CCD4, PSY1, *β*-HOASE, NCED2, and NCED3, which influence the flower color formation. A network analysis implicated MYB4 and MYB85 in regulating the phenylalanine pathway, flavonoid pathway, xanthophylls pathway, and carotenoids pathway. These different pathways may work together through associations to influence the formation of flower colors. In Arabidopsis, *AtPAP1*, *AtPAP2*, *AtMYB113*, and *AtMYB114* are involved in the flavonoid metabolic pathway by regulating the genes of the flavonoid metabolic pathway [27,45]. The *AtTT2* encodes the R2R3-MYB transcription factor that can form a ternary complex (MBW complex) with TT8 (bHLH) and TTG1 (WD40 protein), which regulates the accumulation of proanthocyanidins to some extent [45]. *BnaC03.MYB4*, orthologous to the Arabidopsis gene *ATMYB4*, encodes a R2R3 MYB protein, which plays a role in abiotic stress [46] and lignin synthesis pathways [47]. *BnaC03.MYB85*, orthologous to the Arabidopsis gene *ATMYB85*, directly activates lignin biosynthesis genes and phenylalanine biosynthesis genes during secondary wall formation [48]. However, how the TFs MYB4 and MYB85 regulate the genes of the carotenoid pathway and affect flower color remains to be explored in *B. napus*.

The microRNAs (miRNAs) play important roles in plant growth, development, and response to stress [49,50,51,52]. It is indicated that miRNAs are also associated with petal color formation [53,54]. In the petals of strawberry (*Fragaria* × *ananassa*), it was found that the FamiRNA828a and FamiRNA858_R-2 were targeted by *FaMYB114* and *FaMYB308*, respectively. For flower color intensity, this suggested that FamiR828a is negatively correlated with anthocyanin accumulation in pink-flowered strawberry petals [53]. It was also found that the mcr-miR858-MYB1/MYB5 and mcr-miR396-*McCHI* were involved in inhibiting anthocyanin synthesis in *Malus crabapple* [54]. In the present study, some MYB TFs, such as BnaC03.MYB4, BnaC03.MYB73, BnaC03.MYB98, BnaC03.MYB102, and BnaC03.MYB85, were also found in our multi-omics analysis. However, the relationship between miRNAs and these MYB TFs needs further investigation.

## 4. Materials and Methods

### 4.1. Plant Materials

All plant materials were planted in an experimental field (Wuhan, Hubei Province of China), and the standard management described before was followed [55]. The WP *B. napus* 16214 was provided by the Hybrid Rape Research Center of Shaanxi Province, and the YP *B. napus* 16287 was discovered in our routine field trials. A reciprocal cross was conducted between the *B. napus* of 16214 (WP) and 16287 (YP), and F_1_ hybrids were obtained. The phenotype of F_2_ individuals was also inspected, and the materials with a series of petal colors (including orange-yellow, yellow, light-orange, creamy-white, and white) were observed.

### 4.2. BSA Analysis of Two Pools of WP and YP in the F_2_ Population

A total of 217 F_2_ individuals were investigated, of which 30 individuals with WP and YP were selected for BSA. The genomic DNA of selected individuals was extracted using a Plant Genome DNA Extraction Kit (Tiangen, Beijing, China) and balanced–mixed to construct the two pools of WP and YP. The WP and YP then underwent next-generation sequencing (NGS, MGISEQ-2000RS, MGI Tech Co., Ltd., Shenzhen, China) together with the genomic DNA of 16214 and 16287, respectively.

The raw data were filtered and mapped to the Darmor_*bzh B. napus* genome using Bowtie2 software [56]. The significant loci were mapped to the ZS11 genome with a higher sequenced quality ZS11 genome released [57]. The SNPs and InDels between 16214 and 16287 were filtered using Genome Analysis Toolkit software [58], and the SNP index as well as the Δ(SNP-index) of the WP and YP were calculated subsequently. The AGR was defined according to the average value of Δ(SNP-index), the sliding window was set as 1 Mb, and the step size was set as 1 kb. A threshold significance of 0.99 was selected.

### 4.3. Identification of Petal Color-Related Candidate Genes and Potential Regulatory Network

The genetic linkage map can be aligned to the ZS11 genome using BLAST according to the previous study [59]. Based on the collinearity relationship between the genetic linkage map and the ZS11 genome, genes within the AGR were regarded as potential candidate genes.

The orthologous of potential candidate genes in AGR and their annotations were obtained using BLASTN based on the *A. thaliana* database (http://www.arabidopsis.org/) that accessed on 1 November 2023. The interaction network was analyzed and generated using String_V12.0 (http://string-db.org/) that accessed on 1 November 2023 and visualized using Cytoscape_V3.8.2 [60].

### 4.4. RNA-Seq and DEGs Analysis of the WP and YP

The WP and YP at three developmental stages, four days before flowering (S1), two days before flowering (S2), and the day of flowering (S3), were collected for transcriptomic analysis, and three biological replicates were conducted. YP-S1, YP-S2, and YP-S3 represent the RNA sequencing results of S1, S2, and S3 in the YP, respectively. WP-S1, WP-S2, and WP-S3 represent the RNA sequencing results of S1, S2, and S3 in the WP, respectively.

The total RNA was extracted from the petals of WP-S1, WP-S2, WP-S3, YP-S1, YP-S2, and YP-S3 using a RNAprep Pure Plant Plus Kit (TIANGEN, Beijing, China, DP441) with three biological replicates, respectively. The mRNA was isolated from total RNA through an A-T base pairing of magnetic beads with Oligo (dT) to ployA. The mRNA was randomly broken and small fragments of about 300 bp were isolated via magnetic bead screening. The first strand of cDNA was synthesized in the M-MuLV reverse transcriptase system, and then RNaseH was used to degrade the RNA strand. The second strand of cDNA was synthesized in the DNA polymerase I system. The purified double-stranded cDNA was end-repaired, A-tailed, and connected to sequencing adapters. AMPure XP beads (BECKMAN, USA) were used to screen cDNA of about 250–300 bp, PCR amplification was performed, and AMPure XP beads were used again to purify the PCR products finally to obtain a library. The library was constructed using a NEBNext^®^ Ultra^™^ RNA Library Prep Kit (USA) for Illumina^®^. After the library passed the inspection, different libraries were pooled according to the effective concentration and target off-machine data volume requirements, and then Illumina sequencing was performed [61]. Initially, raw data were subjected to quality control to eliminate the low-quality reads using the fastp software [62]. To determine the genes responsible for the randomly fragmented mRNA fragments, the quality-controlled clean reads were aligned to the ZS11 genome. This alignment was carried out according to a previous methodology using the HISAT2 software [63]. Following the alignment, gene expression quantification was performed using StringTie and Ballgown software [63]. This step yielded expression levels for each sample in terms of fragments per kilobase of transcript per million mapped reads (FPKM). Genes were considered differentially expressed if they met the criteria of a False Discovery Rate (FDR) < 0.01 and a log_2_ (Fold Change) ≥ 1. The R package clusterProfiler was used to conduct GO and KEGG enrichment analyses [64].

### 4.5. The Expression Validation of DEGs via RT-qPCR

A HiScript III 1st Strand cDNA Synthesis Kit (Vazyme, Nanjing, China, R312) was used to synthesize the first strand of cDNA from the RNA. Each PCR reaction was carried out three technical times, and the reaction system was performed following the instructions with SYBR qPCR mix (Vazyme, Q712). *BnaActin7* was used as a reference for normalization and analyzed using the QuantStudio 3 Real-Time PCR System (Thermo Fisher, USA), and each reaction was performed in triplicate. The method of 2^−ΔCT^ was used to measure the gene expression according to a previous study [65]. Primers for RT-qPCR are shown in Table S9.

### 4.6. The Method for Carotenoid Measurement

Petals of white, creamy-white, and orange-yellow flowers were freeze-dried, and 0.02 g of petal tissue was taken for carotenoid content measurements. The carotenoid extractions and measurements were performed using the Plant Carotenoid Content Assay Kit (Solaibio, Beijing, China, BC4330). The absorbance of petal extracts was measured at 440 nm. A two-way ANOVA analysis of variance was used to assess the differences in carotenoid content. The GraphPad Prism_V9 was used for this analysis.

### 4.7. The Molecular Marker Design and the Detection of B. napus with WP and YP

The whole-length sequence of *BnaC03.CCD4*, including its 2 kb promoter, was amplified from the genomic DNA of 16214 and 16287 for Sanger sequencing. Two pairs of primers were designed within the differential sequence of *BnaC03.CCD4* between 16214 and 16287, which were subsequently applied to PCR using the genomic DNA of 16214 and 16287, respectively. A single band was detected in the PCR production of the genomic DNA of the WP, while no band was detected in that of the YP when using primers of PF1/PR1, while the opposite is the case for the primers of PF2/PR2. Thus, 16214.PF1/16214.PR1 and 16287.PF2/16287.PR2 were selected as molecular markers in this study.

## 5. Conclusions

In conclusion, a significant locus of *AGR-FC.C3* that influenced petal color was identified in our data. In addition to *BnaC03.CCD4*, some novel genes were also identified, such as *BnaC03.MYB4*, *BnaC03.MYB85*, *BnaC03.ENDO3*, *BnaC03.T22F8.180*, *BnaC03.Q8GSI6*, *BnaC03.LSD1*, *BnaC03.MAP1Da*, *BnaC03.MAP1Db*, and *BnaC03G0739700ZS*, which might be important in regulating petal color in *B. napus*. Finally, a pair of molecular markers were developed for flower color breeding in the future.

## Data Availability

The raw data of the BSA and RNA-Seq were submitted to the National Center for Biotechnology Information with accession numbers PRJNA1039561 and PRJNA1039725, respectively. All data were enclosed in the main text and supplementary information files. Any detailed datasets generated during and/or analyzed during the current study are available from the corresponding author upon reasonable request.

## Supplementary Materials

The following supporting information can be downloaded at https://www.mdpi.com/article/10.3390/plants13040507/s1: Table S1. Evaluation statistics of BSA sequencing data. Note: ReadSum: number of reads after filtering, counting the number of pair-end sequences in a unit of four rows. BaseSum: number of bases after filtering, the number of ReadSums multiplied by the length of the sequence; GC (%): the GC content of the sample. Q20 (%): percentage of total bases with a mass value greater than or equal to 20. Q30 (%): percentage of total bases with a mass value greater than or equal to 30; Table S2. Statistics of BSA for mapping reference genome results. Note: Clean reads: Statistics of clean read data file, one unit per four rows, double-ended separately. Mapped (%): the percentage of clean reads that are localized to the reference genome over all clean reads. Properly mapped (%): the bipartite sequenced sequences are localized to the reference genome, and the distances correspond to the length distribution of the sequenced fragments; Table S3. Number of SNPs detected via BSA; Table S4. Number of genome-wide InDels; Table S5. The MYB transcription factor in AGR-FC.C3; Table S6. Statistical results of RNA-Seq; Table S7. The mapping ratios to the genome of RNA-Seq; Table S8. The primer sequences of molecular markers; Table S9. Primers used for the RT-qPCR evaluation of gene expression quantified via RNA-Seq; Figure S1. Categories of transcription factor families in 951 genes of the AGR-FC.C3 interval; Figure S2. The KEGG enrichment of 951 genes of the AGR-FC.C3 interval; Figure S3. Violin plot of gene expression levels; Figure S4. Pearson correlation analysis (A) and principal component analysis (PCA) of each sample of RNA-Seq (B); Figure S5. RT-qPCR validation of transcriptome data. FPKM values represent transcriptome data, and 2−ΔCT represents the expression of the RT-qPCR; Figure S6 The KEGG pathway of DEGs in groupings of “YP-S1 vs. WP-S1” (A), “YP-S2 vs. WP-S2” (B) and “YP-S3 vs. WP-S3” (C); Figure S7. Heatmap of the expressions of 154 genes in the transcriptome intersection; Figure S8. The heatmap of genes in the flavonoid pathway. ANS, anthocyanidin synthase; DFR, dihydroflavonol-4-reductase; F3H, flavanone 3-hydroxylase; UFGT, UDP-glycosyltransferase; UF3GT, UDP-glucose: flavonoid 3-o-glucosyltransferase; Figure S9. SNPs of BnaC03.CCD4 between WP and YP. The SNPs were located at positions 9900 to 10600 of BnaC03.CCD4. The bases in the rectangular box represent SNPs, where white represents the WP genome sequence, and orange represents the YP genome sequence.
