# Supplementary figures and images for: Multi-Omics Analysis Revealed the AGR-FC.C3 Locus of Brassica napus as a Novel Candidate for Controlling Petal Color
Source: Plants (Basel). 2024 Feb 11;13(4):507. doi: 10.3390/plants13040507 (PMC10892695; doi:10.3390/plants13040507)

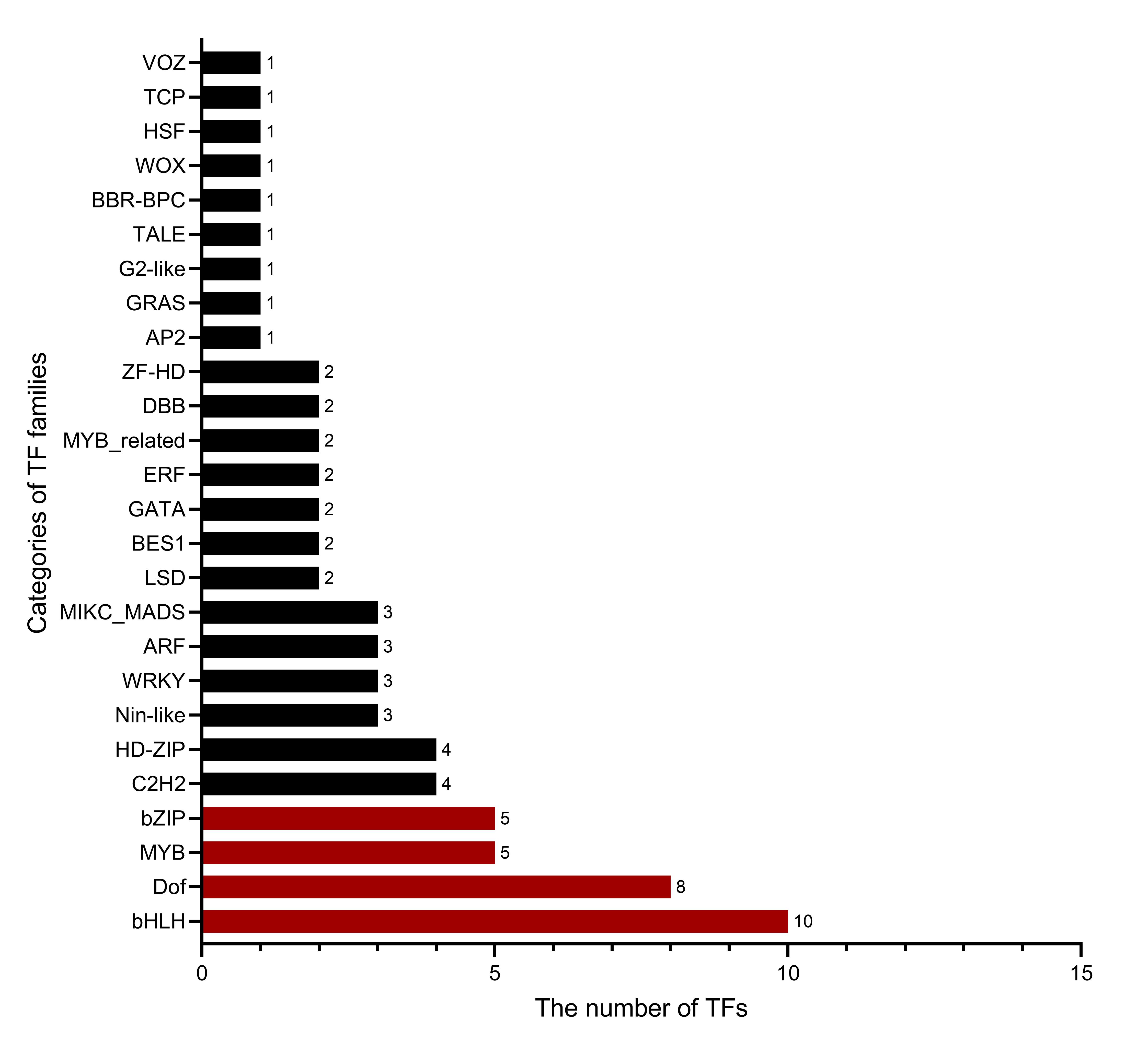

Supplement: Supplementary file 1 [file plants-13-00507-s001.zip › Figure S1.jpg]

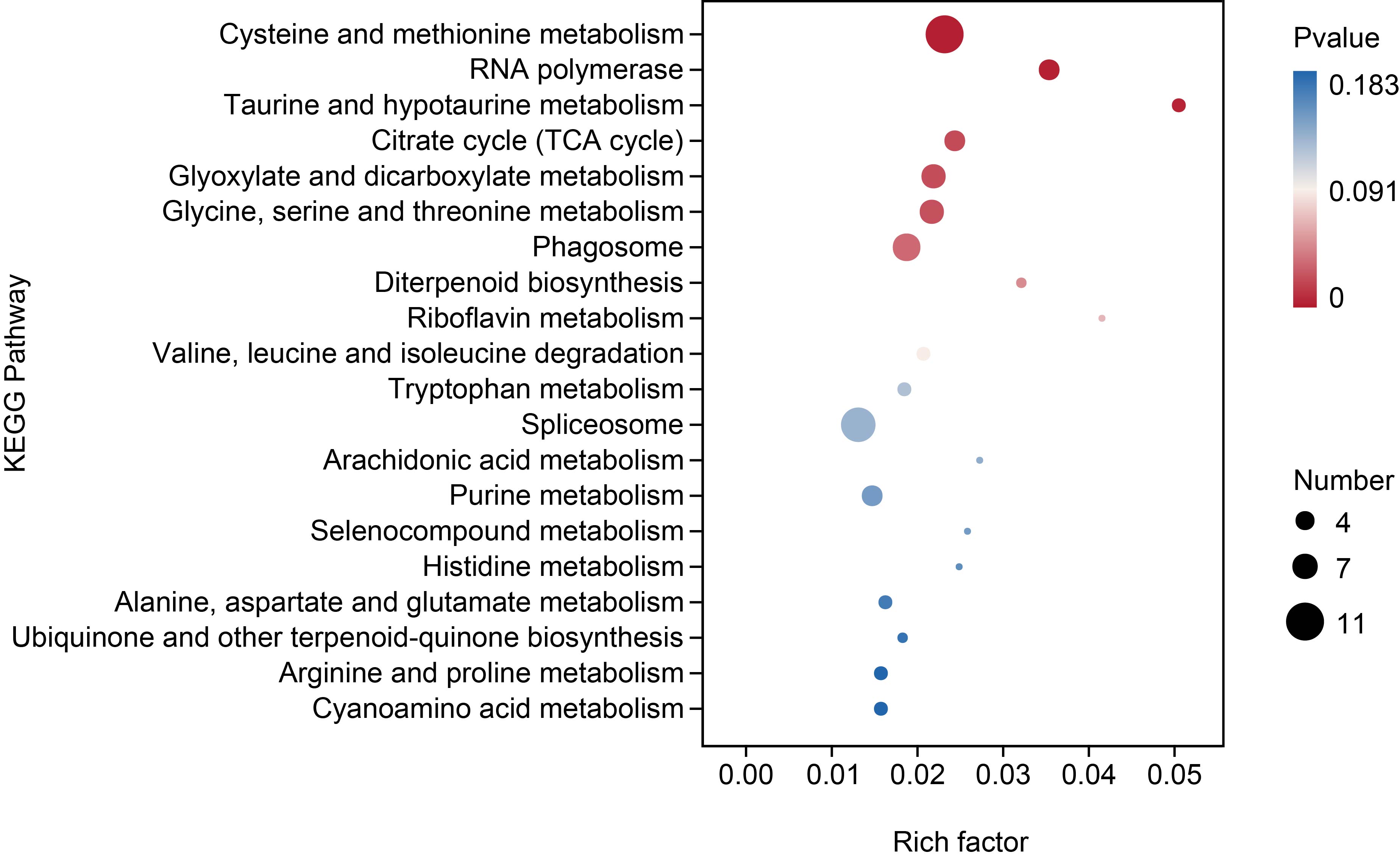

Supplement: Supplementary file 1 [file plants-13-00507-s001.zip › Figure S2.jpg]

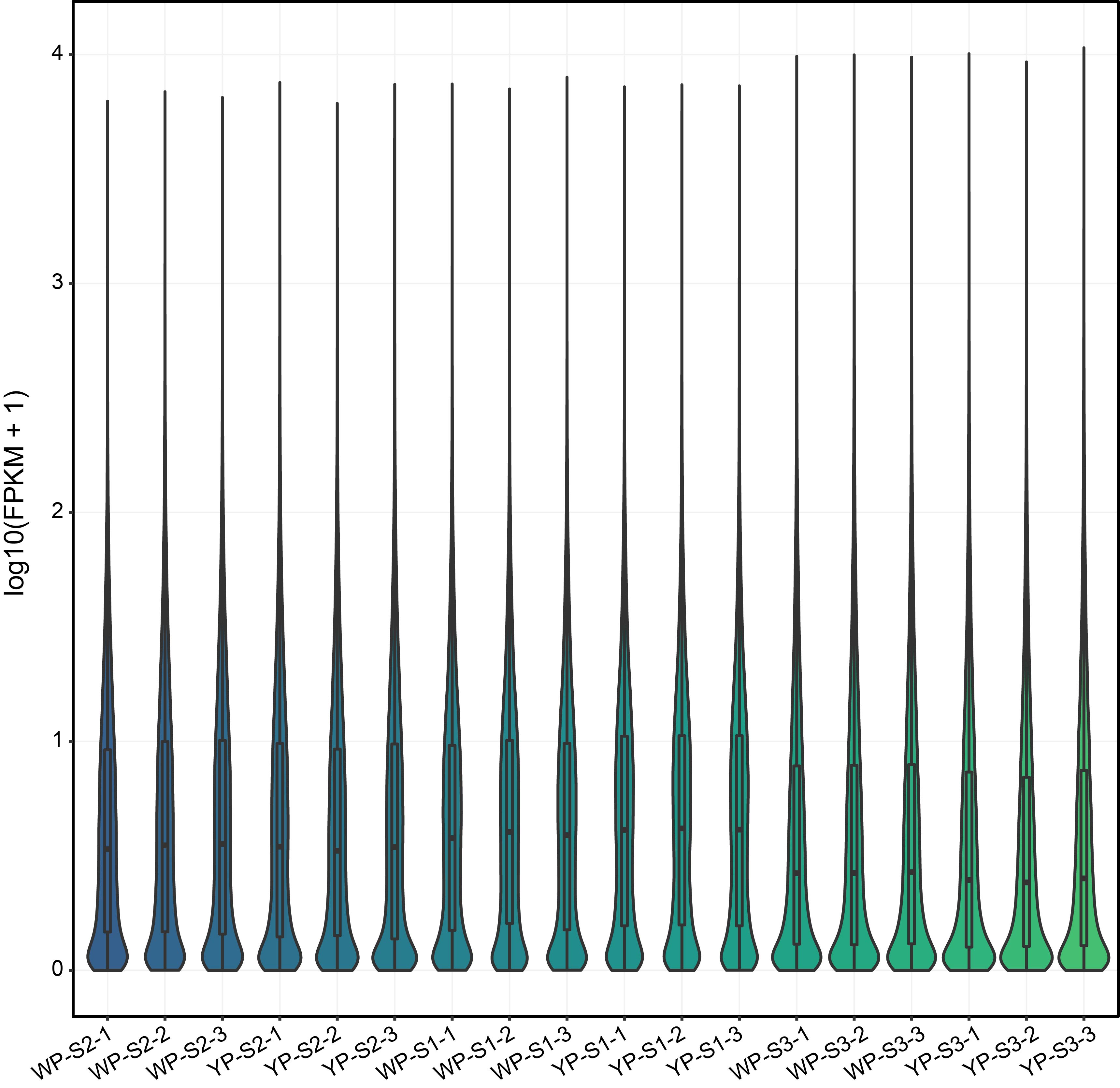

Supplement: Supplementary file 1 [file plants-13-00507-s001.zip › Figure S3.jpg]

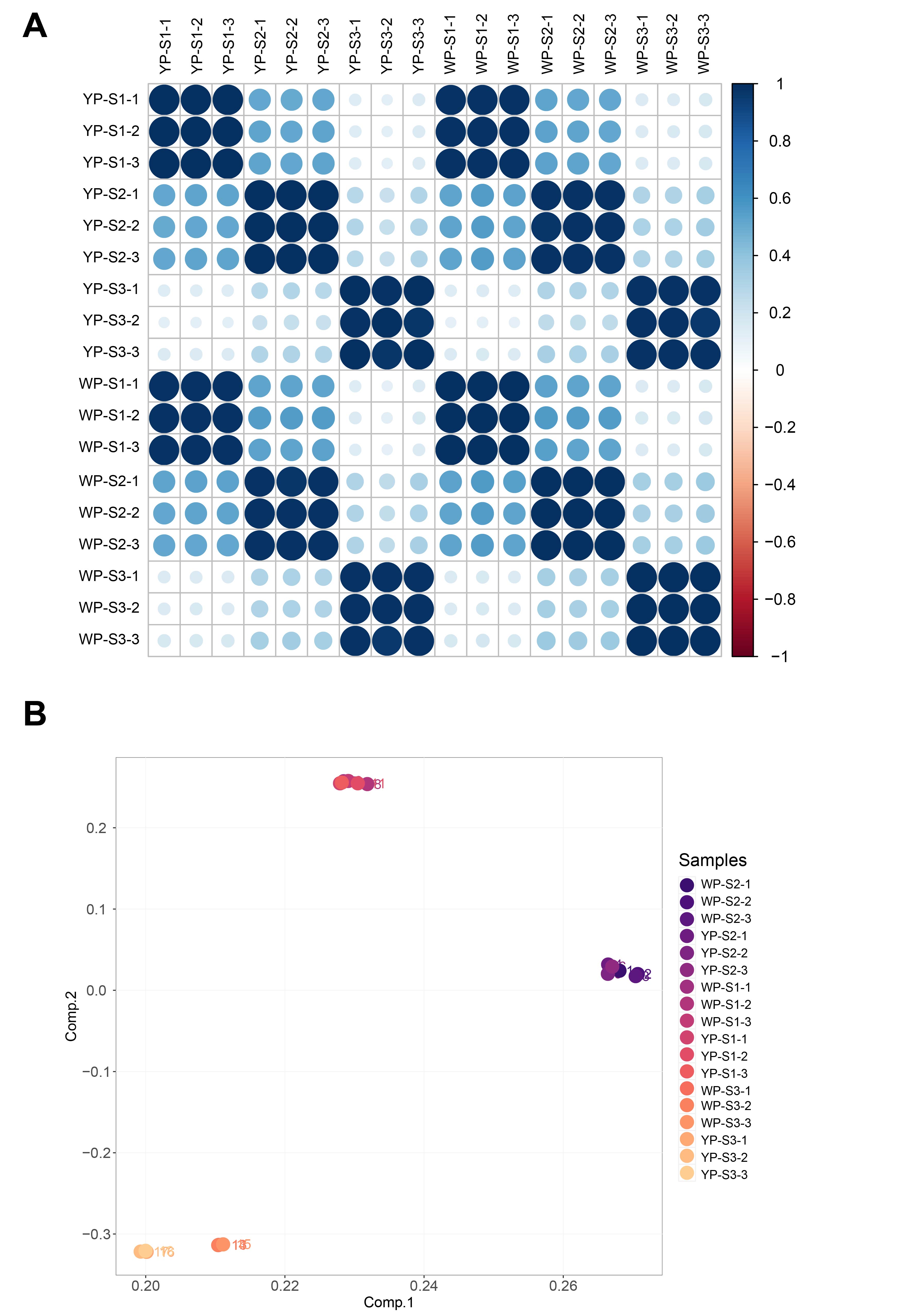

Supplement: Supplementary file 1 [file plants-13-00507-s001.zip › Figure S4.jpg]

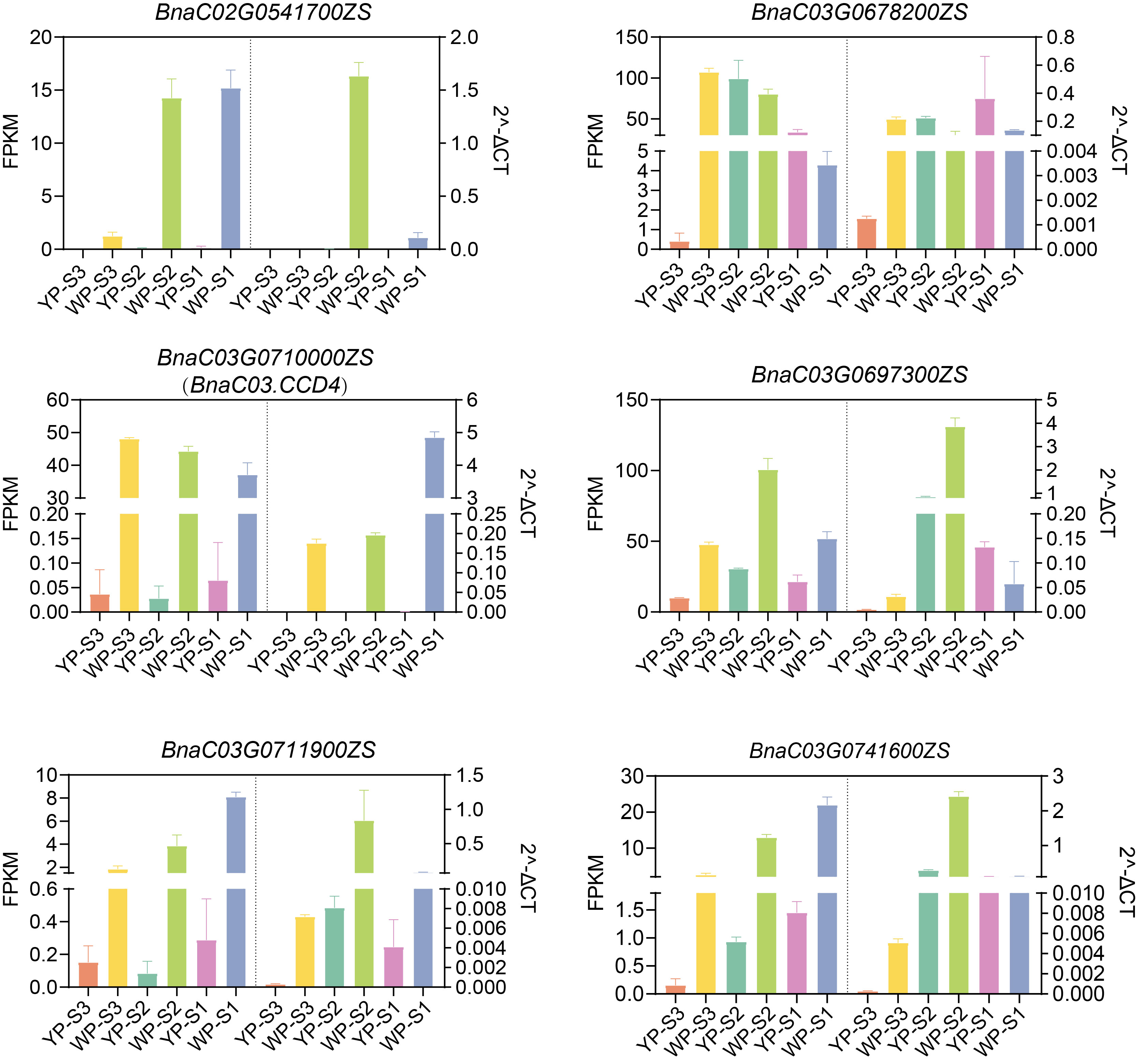

Supplement: Supplementary file 1 [file plants-13-00507-s001.zip › Figure S5.jpg]

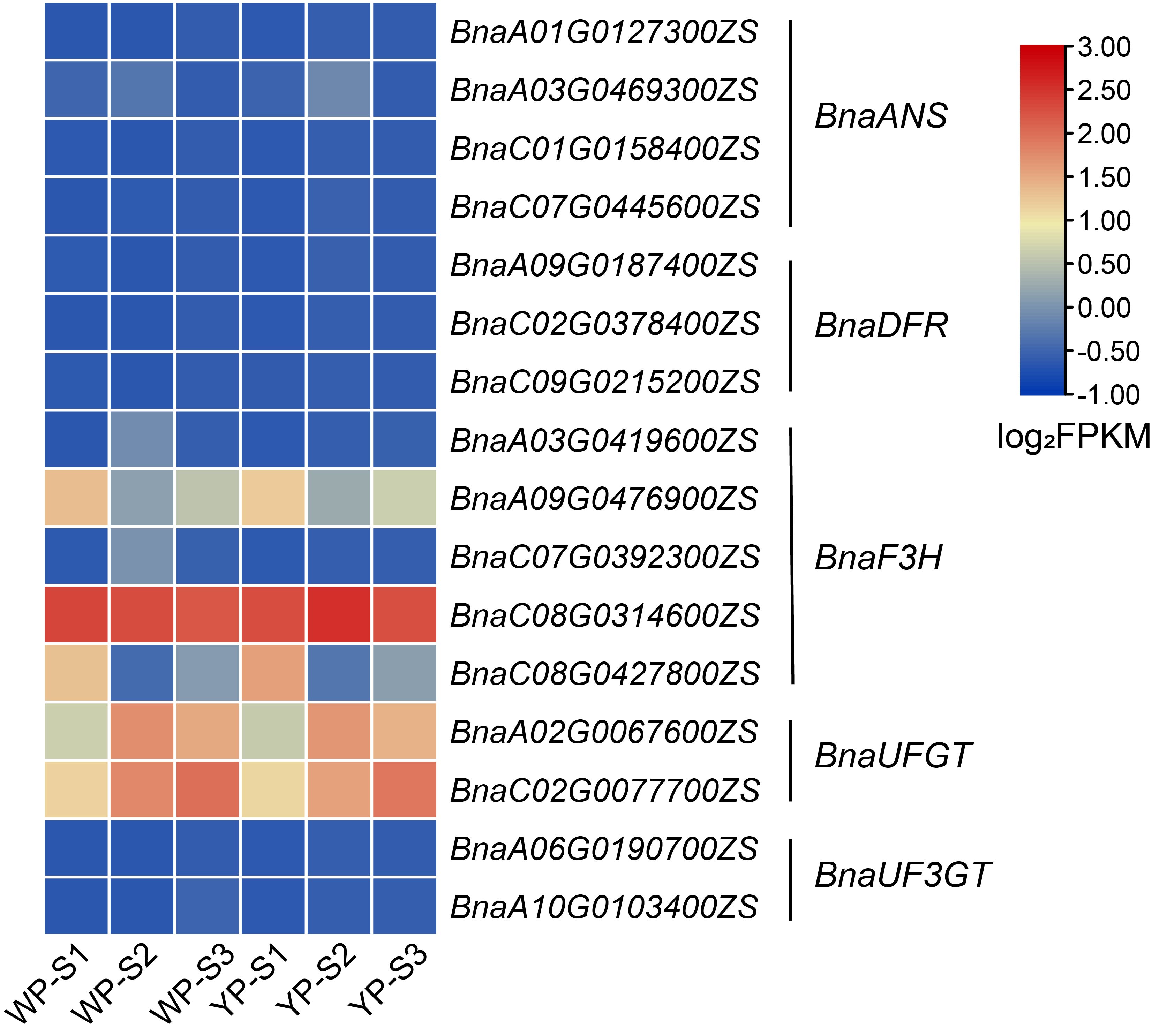

Supplement: Supplementary file 1 [file plants-13-00507-s001.zip › Figure S8.jpg]
